# Blood-Letting in Puerperal Eclampsia

**Published:** 1876-11

**Authors:** John M. Johnson

**Affiliations:** Atlanta, Ga.


ATLANTA

MEDICAL AND SURGICAL JOURNAL

VOL. XIV.] NOVEMBER—1876. [NO. 8

### Original Communications.

### BLOOD-LETTING IN PUERPERAL ECLAMPSIA.

BY J. M. JOHNSON, M.D., ATLANTA, GA.

(*Read before the Atlanta Academy of Medicine.*)

I beg the attention of the Academy of Medicine to the following case of puerperal eclampsia. I report it because I want an expression of opinion on the treatment suggested.

I was called in haste on the —— day of July, at 6 P.M., to see Mrs. E., aged about 30. I had not seen her before, and knew nothing of her case. I found her at confinement, in a terrible fit, and the distress and confusion so great that anything like an intelligible history of the trouble was impossible. But supposing, from what I saw, that she was at confinement, and that I had to deal with a case of puerperal eclampsia, I ordered ice to her head, which was burning hot, and a foot-bath, for her feet were icy cold; sent for chloroform, and proceeded to make a digital examination, not knowing but that she might be near delivery. I found that labor had commenced; the womb was soft and dilatible, and had opened an inch or more. The fit must have lasted full half an hour. The chloroform was brought and administered by inhalation, and the convulsion terminated with the third inspiration.

While the patient was sleeping from the effects of the chloroform, I gathered the following history from her mother and sisters, who are among the most intelligent of our population.

The lady came from Texas, where she resided with her husband, to have her second confinement at her father's house, that she might be under the care of her mother. She had been more delicate with this than with her first pregnancy. For months she had had an unusual thirst, with occasional fevers, attended with progressive swellings of her limbs, body, and face, which was now enormous; that during the day she had suffered dreadfully with headache and total blindness, and had begged piteously for relief.

After sleeping five minutes, she awoke, and sat up in the bed. The foot-bath, administered while sleeping, had warmed her feet, and her head was cooler. Upon questioning her, however, I found her still unconscious, and the next moment she was off in another fit. The chloroform was again applied, and the second fit aborted.

In reply to an inquiry from her parents, as to what further should be done, I proposed blood-letting, which I should have resorted to at once, but had unfortunately left my lancet with another patient. I was in the act of asking a consultation, and of sending for a lancet, when her physician, a homœopathist, entered the room. He thanked me politely for the service I had rendered, and asked me to remain, if I could do so consistently with my views of propriety; which not being able to do, I surrendered the charge and left.

I learned that she had no further convulsions until after the birth of the child, which occurred at 10 P.M., four hours after the attack; but soon after delivery she relapsed again into convulsions, doubtless from metastatic after-pains, (Dr. Power) which continued, as I was informed, until her death, which occurred on the fourth day, while in a state of utter unconsciousness. If there is any error in the history above given, it is because my informers were themselves deceived. I have given the facts substantially as I saw them up to the time I left the case, as well as those derived from other sources.

My object in reporting this case to the Academy is: 1st, because of the great importance of the question to the cause of humanity; 2d, because, as I was informed, the question of blood-letting, as proposed by me, was not adopted; 3d, because the treatment of puerperal eclampsia by blood-letting has become of late an open question, many pathologists contending that it is caused by anæmia, and not by an excess of blood in the brain; and that consequently bleeding tends to increase the death rate, while the abandonment of the practice, and the substitution of the bromides, chloroform and opiates, has lessened it nearly thirty per cent.

In view of all this, I beg the Academy to calmly consider the question, and, after mature deliberation, give its opinion; and let it be given in no uncertain tone.

For more than forty years I have treated puerperal eclampsia with blood-letting, and with invariable success. I have not had a death to occur from it in my practice during this long period; and while I do not always bleed, I generally do so. With most anæmic patients I try other remedies, but if speedy favorable changes do not follow, I resort to the lancet as the "sheet anchor."

Modern physiology comes to the front to-day with new lights, derived from vivisections, and deductions from them, establishing anæmia of the brain as the pathological condition present in eclampsia, to be considered and treated. The means by which this is to be overcome are anæsthetics, sedatives and opiates (!) If this be true, I have now in old age to unlearn the long established therapeutical relations of these remedies, and also the well settled pathology that has been my guide in the treatment of this grave malady.

The study of physiology, and especially nervous physiology, has been wonderfully stimulated within a short period. It began a speculative science, and its voteries seem determined that it shall remain one. Tying the carotids, and exsanguining the brain of a dog, producing death by anæmia of the brain, and producing convulsions resembling eclampsia, it follows that this is the cause of the disease we are considering in the human race. Also that a determination of blood to the brain, sufficient to cause death, does not produce convulsions. It follows, then, that these are the physiological and pathological facts upon which the new lights in the premises rest.

While all this may be true, it proves nothing and disproves nothing. It is in the nervous system that we are to look for the pathogeny of puerperal eclampsia, as well as the pathology of it. Neither repletion nor depletion of the brain is the pathology from which we are to evolve the treatment.

In metastatic labor, where the *vis nervosa* is exerted in other directions than in causing womb contractions, the cord and brain receive the shock, and a convulsion follows. This is called a distributed labor-pain, (Dr. Powers) but it is none the less a puerperal convulsion. This condition continues no longer than true labor contractions are established, but the convulsions return at each pain until the proper effect is produced upon the womb.

In metastatic labor there is not necessarily anæmia of the brain, or irritation of the convolutions of the brain. Nor in albumenuria, or hysteria necessarily so; nor yet irritation of the womb, nor blood poisoning. Here you have inertia of the womb; all of its nerve sympathies are awakened and intensified, but the womb itself is passive. In this form of eclampsia chloroform may give perfect relief. Any nerve sedative, anodyne, or stimulant may do so; in fact, this form of eclampsia may be safely let alone, when the proper action is restored to the womb, and maintained until delivery takes place. But as distributed after-pains may reproduce them, it is safest to bleed, and take all precautions against such result.

Hysteric eclampsia occurs after delivery; generally it is attended with cold surface, especially cold feet and hands, nose and ears, with irregular clonic contractions, attended with sighing and incoherency, but the patient is never unconscious. Here there is anæmia of the brain. Stimulants, foot bathing, mustard, anodynes, etc., will give safe and permanent relief.

Again, eclampsia from irritable womb, with all the phenomena of reflex action, may occur with or without pregnancy. In such cases, the hot douche applied to the os with a Davidson syringe, filling the bowel with hot water, and the free use of hyoscyamus and camphor, bromide of potass., etc., will give relief. Such cases, of course, require an after treatment, but for all the purposes of present relief, at delivery or otherwise, the benefit will be sudden and complete.

Very grave cases of eclampsia arise out of irritation of the cord and gray matter of the brain, involving the basal ganglia; in such cases you have need of all your remedies, including bleeding. Death may ensue from brain hemorrhage or paralysis, or make the patient an invalid for life.

But the gravest form of eclampsia is that attended with anasarca and albumenuria. Beginning a month or two before the full period, with consecutive fever, thirst, scanty urine and general anasarca, and in many instances terminating in effusions within the cavities, and in the cord and ventricles of the brain as well. But if the previous treatment has been judicious, much of the danger will have been obviated before labor sets in; ten or fifteen drops of chloroform given in a swallow of water, three times daily, will clear the urine of its albumenuria, and keep it so (Dr. H. V. M. Miller). This, with half grain doses of saccharated calomel every other night and morning, to maintain secretions, with bromide potass. 20 grains, ℥ss. camphor water, taken together, every hour or two, to relieve headache and nervousness, will in the main be found effective in restoring the functions of the kidneys, and to give much relief otherwise. If it does not, then, in addition, give ten drops tinct. digitalis, with ʒss. tinct. squill, in sweetened water every six hours for two days, and omit one day, and continue to give the portion in this way as long as necessary, will almost certainly relieve the obtunded kidneys, and the anasarca also. But if, under these circumstances, convulsions should intervene, then I would call the lancet into requisition to any extent necessary to bring relief. Blood-letting is, under such circumstances, the best antispasmodic, the best diuretic, and the best sedative one can employ.

The lady in question was swollen, apparently, to the utmost tension, with pupils dilated and eye-balls projecting; the limbs, body, and features so distorted that her friends could not have recognized her. I had no means of ascertaining whether albumenuria was present or not.

To conclude, blood-letting, in such cases as this, restores the functions of the kidneys, promotes absorption from the overfilled cavities, brings relief to the overburdened heart, and also to the perturbed brain and nervous system.

I have very briefly summarised the points presented in this paper. It becomes necessary to do so, as I cannot be present to present my views in person. I beg that the Academy will speak boldly out, and condemn or approve, as judgment, experience, or honest conviction may dictate.

NOTE.—I am indebted to Dr. Henry Frazer Campbell for several important items. I regret not having seen his invaluable paper on Blood-letting in Puerperal Eclampsia, in the August number of the *American Obstetrical Journal*, in time to have been more extensively benefited by it. I saw it by accident just as this paper was completed.

